# Supplementary material for: Selection on plastic adherence leads to hyper-multicellular strains and incidental virulence in the budding yeast
Source: eLife. 2023 Nov 2;12:e81056. doi: 10.7554/eLife.81056 (PMC10764007; doi:10.7554/eLife.81056)
Supplement: Supplementary file 1. — (a) YJM311 Strains used in virulence experiments. (b) YJM128 Strains used in virulence experiments. (c) Results of the mixed-effect linear model for whole-population cell count data over experimental cycles. (d) Results of the mixed-effect linear model for clonal plastic adherence data. (e) Results of the mixed-effect linear model for clonal CCM data. (f) Results of the mixed-effect linear model for clonal flor data. (g) Results of the mixed-effect linear model for clonal PSH data. [file elife-81056-supp1.docx]

| **Strain Name** | **Background**  **(RepPop-Cycle-Clone#)** | **Experimental Identity** | **Multicellular Designation** | **Avg. PSH Index** | **Avg. Flor Score** | **Avg. CCM Score** | **Avg. Fluor. Read.** |
| --- | --- | --- | --- | --- | --- | --- | --- |
| HMY7 | YJM311, *PGK1*-*mCherry-KanMX* | Ancestor | Heterozygous Ancestral Isolate |  |  |  |  |
| HMY596 | C7s-8-8 | Sexual control clone (cycle 8) | Low (L1) | 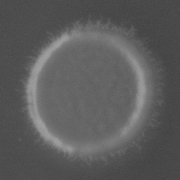26.33 | 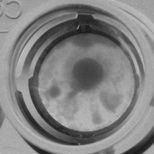0 | 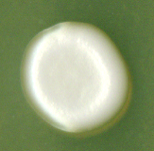1 | 1.174 |
| HMY597 | Anc-0-3 | Ancestral clone | Low (L2) | 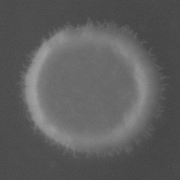24.5 | 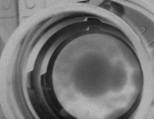0 | 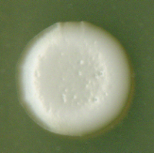1 | 0.939 |
| HMY598 | C7a-8-6 | Asexual control clone (cycle 8) | Low (L3) | 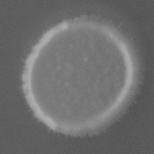16 | 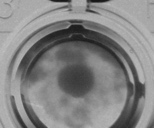0 | 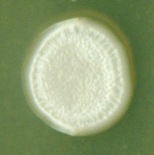2 | 1.573 |
| HMY599 | C7s-8-5 | Sexual control clone (cycle 8) | Low (L4) | 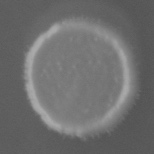18.33 | 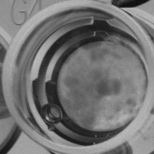0 | 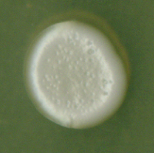1 | 1.204 |
| HMY600 | C7a-2-10 | Asexual control clone (cycle 2) | Low (L5) | 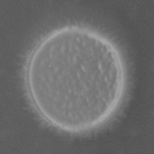16 | 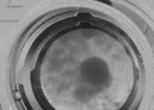0 | 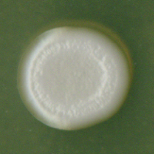1 | 1.233 |
| HMY601 | C7s-2-1 | Sexual control clone (cycle 2) | Low (L6) | 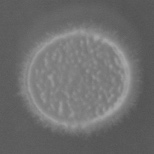26 | 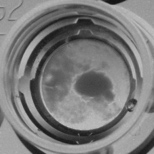0 | 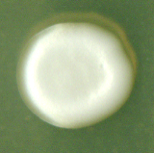1 | 1.475 |
| HMY602 | A7b-8-9 | Asexual experimental clone (cycle 8) | High (H1) | 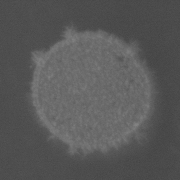26.67 | 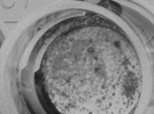2 | 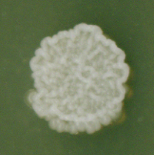4 | 2.082 |
| HMY603 | A7a-8-3 | Asexual experimental clone (cycle 8) | High (H2) | 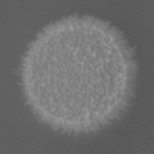31.33 | 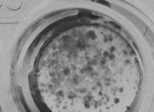1 | 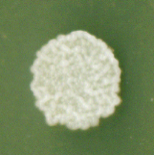4 | 1.454 |
| HMY604 | S7d-8-1 | Sexual experimental clone (cycle 8) | High (H3) | 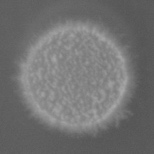38.5 | 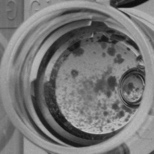1 | 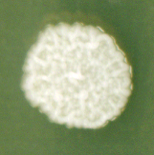4 | 2.095 |
| HMY605 | S7b-8-8 | Sexual experimental clone (cycle 8) | High (H4) | 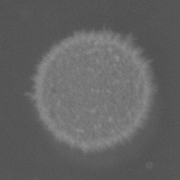20.5 | 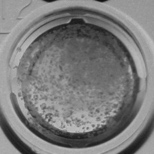2 | 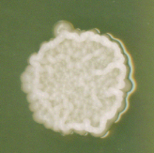3 | 1.925 |
| HMY606 | S7a-8-9 | Sexual experimental clone (cycle 8) | High (H5) | 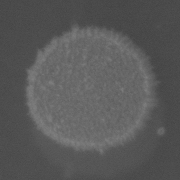18.5 | 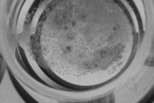2 | 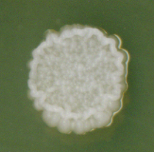3.33 | 1.599 |
| HMY607 | A7d-8-3 | Asexual experimental clone (cycle 8) | High (H6) | 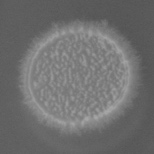22.67 | 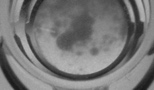0 | 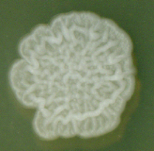5 | 1.259 |

**Supplementary File 1a: YJM311 Strains used in virulence experiments.**

| **Strain Name** | **Background**  **(RepPop-Cycle-Clone#)** | **Experimental Identity** | **Multicellular Designation** | **Avg. PSH Index** | **Avg. Flor Score** | **Avg. CCM Score** | **Avg. Fluor. Read.** |
| --- | --- | --- | --- | --- | --- | --- | --- |
| HMY355 | YJM128, *PGK1*-*mCherry-HygMX* | Ancestor | Heterozygous Ancestral Isolate |  |  |  |  |
| HMY580 | S8d-3-6 | Sexual experimental clone (cycle 3) | Low (L1) | 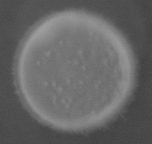  26% | 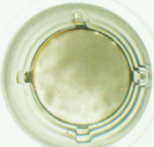0 | 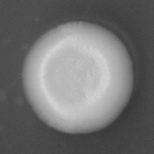1 | 6.04 |
| HMY581 | S8b-1-10 | Sexual experimental clone (cycle 1) | Low (L2) | 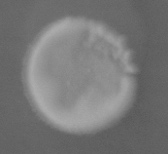  22% | 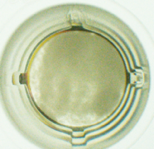0 | 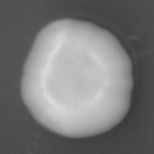1 | 3.44 |
| HMY582 | S8b-3-8 | Sexual experimental clone (cycle 3) | Low (L3) | 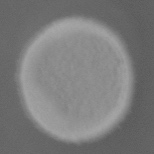  24% | 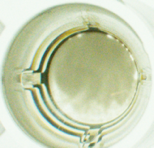0 | 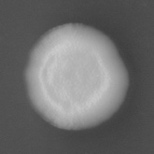1 | 8.78 |
| HMY579 | Anc-8-5 | Ancestral clone | Low (L4) | 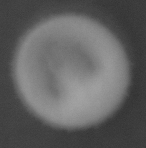  23.34% | 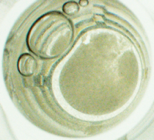1 | 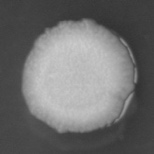2 | 4.66 |
| HMY584 | A8c-3-10 | Asexual control clone (cycle 3) | Low (L5) | 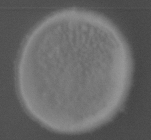  20.67% | 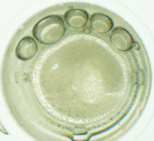2 | 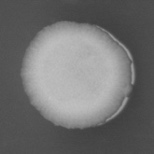1 | 7.5 |
| HMY575 | Anc-8-19 | Ancestral clone | Low (L6) | 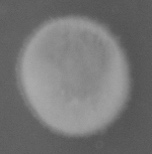  22.67% | 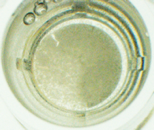1 | 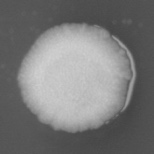2 | 7.3 |
| HMY570 | A8a-6-8 | Asexual experimental clone (cycle 6) | High (H1) | 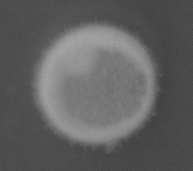  20% | 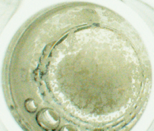3 | 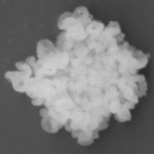5 | 11.06 |
| HMY571 | S8b-6-1 | Sexual experimental clone (cycle 6) | High (H2) | 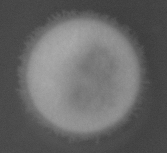  26.5% | 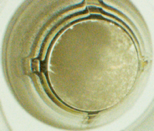1 | 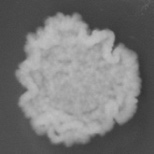3 | 9.04 |
| HMY572 | A8d-9-10 | Asexual experimental clone (cycle 9) | High (H3) | 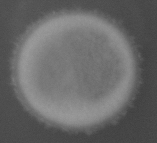  29.67% | 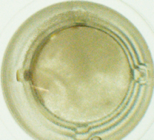0 | 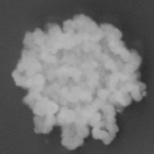3.34 | 9.23 |
| HMY573 | S8b-9-5 | Sexual experimental clone (cycle 9) | High (H4) | 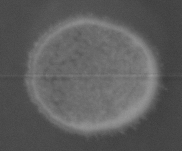  31.67% | 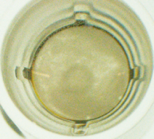1 | 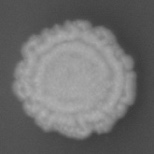2.34 | 6.93 |
| HMY574 | A8d-9-2 | Asexual experimental clone (cycle 9) | High (H5) | 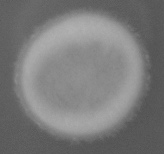  27.33% | 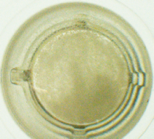1 | 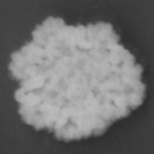3.67 | 6.34 |
| HMY576 | A8a-9-8 | Asexual experimental clone (cycle 9) | High (H6) | 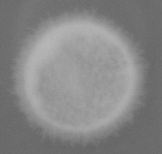  39% | 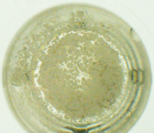3 | 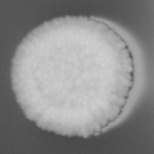2 | 7.79 |

**Supplementary File 1b: YJM128 Strains used in virulence experiments.**

|  | **YJM311** | | | **YJM128** | | |
| --- | --- | --- | --- | --- | --- | --- |
| **Fixed Effects** | coefficient | st. error | t-value | coefficient | st. error | t-value |
| Cycle: Control | -0.05528 | 0.03437 | -1.608 | 0.02182 | 0.09041 | 0.241 |
| Cycle: Sexual | **0.05407** | 0.02433 | 2.223 | **0.46993** | 0.12151 | 3.868 |
| Cycle: Asexual | **0.18435** | 0.02422 | 7.612 | **0.59841** | 0.12747 | 4.695 |
| **Random Effects** | variance | | | variance | | |
| Population(Treatment) | 0.2593 | | | 7.683 | | |
| Residual | 0.9466 | | | 17.302 | | |
| Residual DF | 629 | | | 525 | | |

**Supplementary File 1c: Results of the mixed-effect linear model for whole-population cell count data over experimental cycles.** Cell counts were transformed by adding one and taking the natural log; data were analyzed with the lme4 package in R. Coefficients whose confidence intervals do not encompass zero are bolded.

|  | **YJM311** | | | **YJM128** | | |
| --- | --- | --- | --- | --- | --- | --- |
| **Fixed Effects** | coefficient | st. error | t-value | coefficient | st. error | t-value |
| Cycle: Control | 0.008143 | 0.028509 | 0.286 | 0.01614 | 0.03805 | 0.424 |
| Cycle: Sexual | **0.039812** | 0.020266 | 1.964 | 0.01174 | 0.05184 | 0.227 |
| Cycle: Asexual | **0.100228** | 0.020125 | 4.980 | **0.18686** | 0.05661 | 3.301 |
| **Random Effects** | variance | | | variance | | |
| Population(Treatment) | 0.1482 | | | 2.328 | | |
| Residual | 0.4347 | | | 4.066 | | |
| Residual DF | 391 | | | 574 | | |

**Supplementary File 1d: Results of the mixed-effect linear model for clonal plastic adherence data.** Data were analyzed with the lme4 package in R. Coefficients whose confidence intervals do not encompass zero are bolded.

|  | **YJM311** | | | **YJM128** | | |
| --- | --- | --- | --- | --- | --- | --- |
| **Fixed Effects** | coefficient | st. error | t-value | coefficient | st. error | t-value |
| Cycle: Control | -0.02432 | 0.02783 | -0.874 | 0.007192 | 0.009940 | 0.724 |
| Cycle: Sexual | **0.11899** | 0.01968 | 6.046 | **0.048360** | 0.013161 | 3.675 |
| Cycle: Asexual | **0.13610** | 0.01968 | 6.916 | **0.052230** | 0.014772 | 3.536 |
| **Random Effects** | variance | | | variance | | |
| Population(Treatment) | 0.2748 | | | 0.1702 | | |
| Residual | 0.3588 | | | 0.2789 | | |
| Residual DF | 395 | | | 584 | | |

**Supplementary File 1e: Results of the mixed-effect linear model for clonal CCM data.** Data were analyzed with the lme4 package in R. Coefficients whose confidence intervals do not encompass zero are bolded.

|  | **YJM311** | | | **YJM128** | | |
| --- | --- | --- | --- | --- | --- | --- |
| **Fixed Effects** | coefficient | st. error | t-value | coefficient | st. error | t-value |
| Cycle: Control | 0.006646 | 0.024077 | 0.276 | **-0.10927** | 0.01633 | -6.692 |
| Cycle: Sexual | **0.074519** | 0.017025 | 4.377 | 0.03118 | 0.02161 | 1.443 |
| Cycle: Asexual | **0.162927** | 0.017025 | 9.570 | -0.00291 | 0.02407 | -0.121 |
| **Random Effects** | variance | | | variance | | |
| Population(Treatment) | 0.1678 | | | 0.3527 | | |
| Residual | 0.2780 | | | 0.7740 | | |
| Residual DF | 395 | | | 584 | | |

**Supplementary File 1f: Results of the mixed-effect linear model for clonal flor data.** Data were analyzed with the lme4 package in R. Coefficients whose confidence intervals do not encompass zero are bolded.

|  | **YJM311** | | | **YJM128** | | |
| --- | --- | --- | --- | --- | --- | --- |
| **Fixed Effects** | coefficient | st. error | t-value | coefficient | st. error | t-value |
| Cycle: Control | **-0.6111** | 0.3012 | -2.029 | **1.2908** | 0.1952 | 6.612 |
| Cycle: Sexual | **-0.6474** | 0.2130 | -3.040 | **0.6096** | 0.2572 | 2.370 |
| Cycle: Asexual | **-0.9649** | 0.2156 | -4.475 | **0.9085** | 0.2750 | 3.304 |
| **Random Effects** | variance | | | variance | | |
| Population(Treatment) | 44.81 | | | 21.48 | | |
| Residual | 40.29 | | | 126.08 | | |
| Residual DF | 390 | | | 584 | | |

**Supplementary File 1g: Results of the mixed-effect linear model for clonal PSH data.** Data were analyzed with the lme4 package in R. Coefficients whose confidence intervals do not encompass zero are bolded.
